# Noninvasive quantification of target availability during therapy using paired-agent fluorescence tomography

**DOI:** 10.7150/thno.45273

**Published:** 2020-09-14

**Authors:** Boyu Meng, Margaret R. Folaron, Rendall R. Strawbridge, Negar Sadeghipour, Kimberley S. Samkoe, Kenneth Tichauer, Scott C. Davis

**Affiliations:** 1 Thayer School of Engineering, Dartmouth College, Hanover, NH 03755.; 2 Biomedical Engineering, Illinois Institute of Technology, Chicago, IL 60616.; 3 Geisel School of Medicine, Dartmouth College, Hanover, NH 03755.

**Keywords:** quantitative molecular imaging, fluorescence tomography, receptor-targeted therapy, antibody treatment monitoring, receptor imaging

## Abstract

Immuno-oncological treatment strategies that target abnormal receptor profiles of tumors are an increasingly important feature of cancer therapy. Yet, assessing receptor availability (RA) and drug-target engagement, important determinants of therapeutic efficacy, is challenging with current imaging strategies, largely due to the complex nonspecific uptake behavior of imaging agents in tumors. Herein, we evaluate whether a quantitative noninvasive imaging approach designed to compensate for nonspecific uptake, MRI-coupled paired-agent fluorescence tomography (MRI-PAFT), is capable of rapidly assessing the availability of epidermal growth factor receptor (EGFR) in response to one dose of anti-EGFR antibody therapy in orthotopic brain tumor models.

**Methods:** Mice bearing orthotopic brain tumor xenografts with relatively high EGFR expression (U251) (N=10) or undetectable human EGFR (9L) (N=9) were considered in this study. For each tumor type, mice were either treated with one dose of cetuximab, or remained untreated. All animals were scanned using MRI-PAFT, which commenced immediately after paired-agent administration, and values of RA were recovered using a model-based approach, which uses the entire dynamic sequence of agent uptake, as well as a simplified “snapshot” approach which requires uptake measurements at only two time points. Recovered values of RA were evaluated between groups and techniques. Hematoxylin & eosin (H&E) and immunohistochemical (IHC) staining was performed on tumor specimens from every animal to confirm tumor presence and EGFR status.

**Results:** In animals bearing EGFR(+) tumors, a significant difference in RA values between treated and untreated animals was observed (RA = 0.24 ± 0.15 and 0.61 ± 0.18, respectively, p=0.027), with an area under the curve - receiver operating characteristic (AUC-ROC) value of 0.92. We did not observe a statistically significant difference in RA values between treated and untreated animals bearing EGFR(-) tumors (RA = 0.18 ± 0.19 and 0.27 ± 0.21, respectively; *p* = 0.89; AUC-ROC = 0.55), nor did we observe a difference between treated EGFR(+) tumors compared to treated and untreated EGFR(-) tumors. Notably, the snapshot paired-agent strategy quantified drug-receptor engagement within just 30 minutes of agent administration. Examination of the targeted agent alone showed no capacity to distinguish tumors either by treatment or receptor status, even 24h after agent administration.

**Conclusions:** This study demonstrated that a noninvasive imaging strategy enables rapid quantification of receptor availability in response to therapy, a capability that could be leveraged in preclinical drug development, patient stratification, and treatment monitoring.

## Introduction

There is now widespread recognition that immuno-oncological treatment strategies that target patient-specific tumor vulnerabilities and/or attempt to affect the complex interactions between tumor cells and the immune system will have an increasing impact on cancer care 1-4. However, while subpopulations of patients are highly responsive, overall response rates are often low 5, 6, suggesting there is a substantial gap between laboratory understanding and the deployment of these treatment strategies in patients 7. This is at least in part a consequence of an inability to both noninvasively assess the availability of drug targets and determine whether the administered drug engages the targets as expected 7-10. Enabling these types of evaluations in both preclinical animal models and patients would have a substantial impact on translational efforts for new drugs, stratification of patients to different treatment regimens, and regular monitoring for reevaluation of drug engagement during treatment.

The development of molecular imaging strategies using targeted agents has often been viewed as a promising avenue for assessing drug targets noninvasively 11, 12. Yet, the irregular and variable vasculature in solid tumors presents a persistent challenge for isolating and quantifying receptor availability in these pathologies 8, 13-15. Specifically, the rates at which the agent transports between the vascular and extra-vascular spaces, and the rates at which it binds and becomes dissociated from the receptor, among other factors 16-19, all play a role in determining total agent accumulation. As a consequence, images of targeted contrast agents in solid tumors are often composed of signal from bound agent and an unpredictable accumulation of unbound agent. Recent efforts to isolate binding using noninvasive fluorescence lifetime changes have shown promise 20-22; yet the dependence on measuring fluorescence lifetime precludes the approach being deployed using conventional imaging modalities, and response to therapy has not yet been reported using these techniques. Analysis of tissue specimens is an obvious alternative, though assessing *in vivo* receptor availability using these techniques is problematic because they are prone to tissue sampling error, do not permit longitudinal assessments, and do not inform on critical factors such as systemic and local delivery of the agents. Accordingly, these techniques are not generally accurate representations of the receptors available for binding *in vivo*. In this context, accurate, rapid assessment of target availability *in vivo* has remained a challenge.

In an effort to overcome these barriers, we have previously reported on a paired-agent imaging strategy designed to compensate for non-specific uptake of targeted imaging agents to produce quantitative estimates of receptor availability (RA), also known as “binding potential” 23. The recovery of this parameter is enabled by imaging the early dynamics of two imaging agents, a targeted agent and an untargeted isotype, usually administered simultaneously. The RA parameter, defined as the product of the concentration of receptor targets *available* for binding and the affinity of the agent to the target, is then estimated by fitting the kinetic curves of the two agents to a dual-agent compartment model 23, 24. In this construct, the uptake behavior of the untargeted agent is used to compensate for the non-specific uptake behavior of the targeted agent, enabling isolation of RA. Using this strategy, we previously reported that dynamic MRI-coupled paired-agent fluorescence tomography (MRI-PAFT) was capable of estimating the concentration of epidermal growth factor receptor (EGFR) available for binding in EGFR(+) orthotopic glioma models 25. Although these results suggested that the noninvasive paired-agent methodology may be capable of revealing the effects of targeted therapy on RA, this has not yet been reported.

We hypothesize that noninvasive paired-agent imaging applied using MRI-PAFT is capable of rapidly estimating RA in response to receptor-targeted therapy. Specifically, we evaluated the response of MRI-PAFT to one dose of anti-EGFR antibody in tumors with different innate receptor status. Receptor availability was recovered using two methodologies; namely, the *model-fitting* approach, which estimates RA by fitting a compartmental model to the full hour-long paired agent dynamic sequence data, and the *snapshot* approach which simplifies the model into a ratio expression using just two measurement time points. To evaluate these methods, we quantified and compared; (1) RA between treated and untreated mice bearing either EGFR(+) or EGFR(-) tumors using both the model-fitting and snapshot techniques, (2) RA between EGFR(+) and (-) tumor groups using both techniques, (3) single agent uptake in treated and untreated EGFR(+) and (-) tumors at both short and long post-agent-administration times, and assessed (4) the ability of the snapshot RA technique to estimate the model-based RA values. Pathological analysis, consisting of hematoxylin & eosin (H&E) and immunohistochemical (IHC) staining, was performed on tumor specimens from every animal to confirm tumor presence and EGFR status.

## Materials and Methods

### Experimental Design

**Figure 1:**
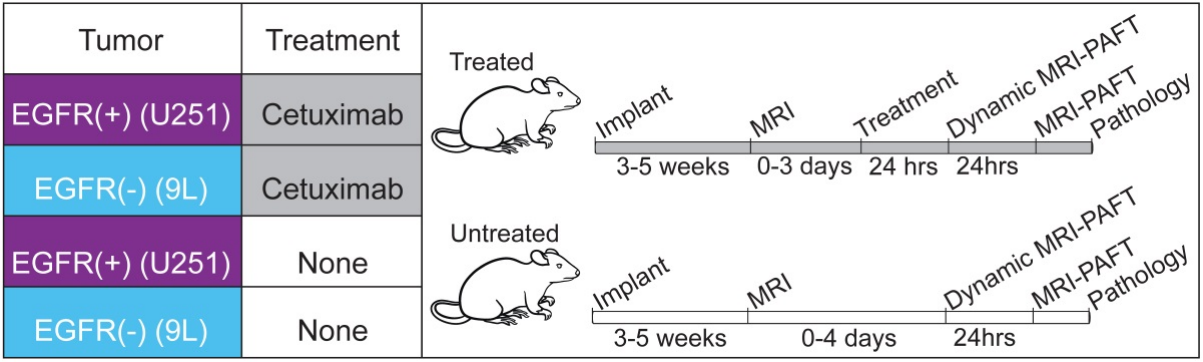
**Experimental design to evaluate MRI-PAFT for receptor quantification in response to therapy.** Four groups of mice were used, two groups implanted with EGFR(+) tumors (U251 human glioma) and two implanted with EGFR(-) tumors (9L rat sarcoma). Treated animals were administered one human equivalent dose of cetuximab 24 h prior to dynamic imaging. All mice were scanned with dynamic MRI-PAFT for receptor quantification and then imaged again with a single MRI-PAFT scan 24 h later. Immediately after the 24-h scan, animals were euthanized for H&E and IHC analysis.

We evaluated the capacity of MRI-PAFT to assess EGFR availability in response to receptor targeted therapy using groups of treated and untreated mice bearing orthotopic brain tumor xenografts. As described in the experimental framework provided in Figure 1, the study considered tumors known to have high expression of EGFR (U251 human glioma) as well as tumors with low expression levels of the receptor (9L rat gliosarcoma), included as additional biological controls 26-28. We applied the paired-agent strategy using a cocktail consisting of ABY029, a GLP version of the anti-EGFR Affibody molecule labeled with LI-COR IRDye 800CW, and the Affibody molecule negative control molecule labeled with LI-COR IRDye 680RD as the untargeted isotype. Animals in the treated group were administered one human-equivalent therapeutic dose of cetuximab, a clinical-grade anti-EGFR antibody, 24 h prior to MRI-PAFT scanning. This high-affinity antibody occupies the binding site used by the Affibody peptide, and thus renders the receptor unavailable to the targeted imaging agent 27, 29. During the course of the study one animal showed evidence of a large hematoma occupying about ¼ of the brain in MRI (confirmed with follow-up pathology) and was excluded from the analysis. In the final analysis, all groups consisted of five animals, except for the treated 9 L tumor group, which consisted of four animals.

### Animal Models

All procedures involving animals were conducted in accordance with protocols approved by the Institutional Animal Care and Use Committee (IACUC) at Dartmouth College. The models used in this study consisted of EGFR(+) (U251 human glioma) or EGFR(-) (9L rat sarcoma) tumor xenografts implanted intracranially in athymic nude mice. Animals were purchased from an approved vender (Charles River Laboratories, Wilmington, MD) at 6-8 weeks of age, housed in a standard cage with 12-hour dark and light cycle, and given free access to food and water. To initiate tumor growth, animals were inoculated with 10^6^ tumor cells through a burr hole in the skull anterior to the bregma and lateral to the midline of the brain, as described elsewhere 30. Mice receive 5mg/kg Ketoprofen subcutaneously prior to surgery and a second dose 24 h after recovering from surgery. Animals were monitored for health and tumor growth was assessed using gadolinium contrast-enhanced MRI (Gd-MRI) starting two weeks after tumor implantation. A total of 20 animals were enrolled in the study, and randomly assigned into 4 cohorts.

### Imaging agents

The paired imaging agents used in this study consisted of an EGFR-targeted agent and an untargeted isotype. The targeted agent used was ABY-029, a GLP formulation of Affibody's anti-EGFR protein molecule (Affibody AB, Solna, Sweden) conjugated to IRDye 800CW Maleimide (LI-COR Biosciences, Inc., Lincoln, Nebraska). This GLP formulation was produced by Bachem AG as detailed elsewhere 30. The untargeted counterpart was Affibody Imaging Agent, Negative Control bound to LI-COR's IRDye 680RD, also via maleimide conjugation as described elsewhere 31. Conjugation was confirmed using absorption spectrometry and dosing based on protein concentration. For dynamic MRI-PAFT scanning, a solution containing 1 μM of each optical agent and 50 μM of Gadovist (Bayer, Leverkusen, Germany) was prepared.

### MRI-PAFT instrument and image reconstruction

**Figure 2:**
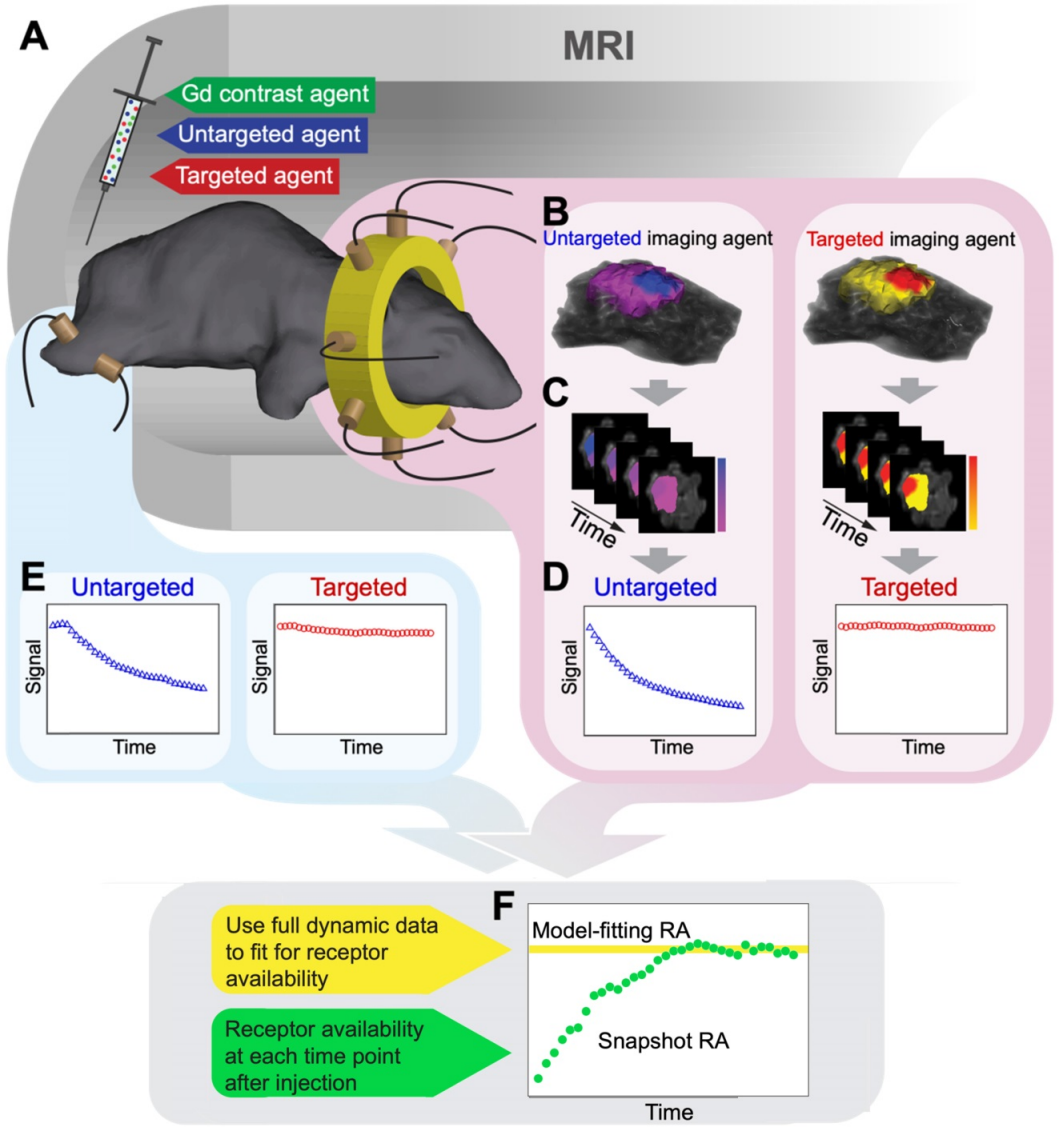
** MRI-PAFT procedure for estimating receptor availability.** (A) Illustration of the MRI-PAFT animal interface inside the magnet bore showing the tomographic fiber array encircling the head, and a pair of optical fibers on the leg for acquiring normal tissue kinetics. (B) Representative volumetric images of fluorescence activity (one frame) in the brain and tumor for both targeted and untargeted agents. Volumes such as these were acquired at approximately 0.5 Hz over the course of over 60 min, resulting in dynamic image stacks of each agent (C). Fluorescence activity was then extracted from the tumor and normal tissue to produce dynamic uptake curves, as shown in (D) and (E), respectively. Data from these curves were then used to determine RA using the model-fitting and snapshot approaches, as illustrated in (F).

The operation of the MRI-coupled fluorescence tomographic instrument has been described in previous publications 25, 32. As used in this study, the instrument consisted of a custom MR/optical rodent RF coil that positions eight optical fibers in a circular array around the head of the mouse and two fibers on the leg, as shown in Figure 2A. Each optical fiber bundle extends out of the MRI room and bifurcates; one branch connecting to a laser source multiplexer and the other to one of 10 spectrometers with motorized filter wheels and low noise CCD camera sensors. During acquisition, the excitation light is automatically sequenced to each fiber while the remaining fibers operate in detection mode. Thus, one tomographic scan of the head consists of 56 fluorescence emission/excitation projections through the head of the animal, each of which is a spectrally-resolved fluorescence emission spectrum. In this study, each dynamic frame consisted of two tomographic scans; one for the untargeted agent (IRDye 680RD bound to Affibody negative control, excited by a 635 nm laser diode) and the other for the targeted agent (ABY-029 excited by a 730 nm laser diode), plus measurements through the leg for each dye, resulting in a total of 114 projections for a paired-agent imaging frame. In some cases, near-source detectors were omitted due to detector saturation.

Each projection measurement is a highly-resolved fluorescence spectrum composed of the emission of the excited agent and tissue autofluorescence. Using basis spectra of the agent alone (acquired in phantoms) and tissue-specific autofluorescence (acquired for each projection in each animal immediately prior to agent administration) a spectral fitting algorithm was used to decouple the agent fluorescence from the background signal 25. The fitted spectrum of the agent was then integrated to produce one intensity value for each projection, calibrated with excitation measurements in the same channel, and then used for image reconstruction.

The MRI-coupled fluorescence tomography image recovery framework used herein has been described extensively elsewhere 33-35. The high resolution Gd-MRI images acquired 15 min after agent administration were segmented into three tissue regions: the head, normal brain, and tumor, using the latest version of the NIRFAST software package 36, 37, built on the 3D Slicer platform. Segmentation involved the use of thresholding, hole filling, and region growing tools followed by manual edits to complete the process. This segmented volume then served as a template upon which the optical data were reconstructed using a hard constraint spatial priors approach 25, 38-40. Specifically, the difference between the measured fluorescence projection data and data calculated from the diffusion approximation of light transport through tissue was minimized to estimate fluorescence activity (the product of the agent's quantum yield and absorption coefficient), using NIRFAST. This process was repeated for each of the two agents at all frames in the dynamic sequence. Example volumes of fluorescence activity overlaid on MRI volumes are shown in Figure 2B.

### Dynamic MRI-PAFT experimental procedure

Three weeks after tumor implantation, mice were monitored for tumor growth using gadolinium-enhanced MRI (Gd-MRI) in a Philips Achieva 3.0T scanner. Once tumors were observed to be larger than 2 mm across, as determined by Gd enhancement in the vicinity of the inoculation, mice were put on study and randomized into either untreated or treated groups. This was repeated for both EGFR(+) and EGFR(-) tumor lines. Mice in the treated group were administered one human equivalent dose of cetuximab (1 mg total using a 2 mg/mL administration in the intraperitoneal cavity 26, 41-43) 24 h before dynamic MRI-PAFT scanning, a time interval commonly used to examine binding effects 15, 44, 45.

The procedures for image acquisition and data analysis applied to each animal are illustrated in Figure 2. In preparation for dynamic scanning, study animals were positioned in the custom RF/optical coil and scout scans were used to ensure tumors were near the plane of the optical fiber array. Before administration of the paired-agent cocktail, a full set of MRI-PAFT scans were acquired, consisting of high resolution T1-weighted MR images (TR=744 ms, TE=9.9 ms, FOV=90 mm, reconstruction matrix = 256*256, slice thickness = 0.75 mm, and slice number = 30) and full optical tomographic scans (and one set of leg measurements) of both agents. To begin acquisition of the dynamic paired-agent data, the study animal was administered the paired-agent cocktail (0.2 nmol of each optical agent mixed with 0.01 mmol of Gadovist (Bayer, Leverkusen, Germany), in a 200 μl volume) into the tail vein, and optical tomographic scanning was initiated immediately thereafter. For each mouse, we acquired 35 frames of data, with each frame consisting of full tomographic scans for both targeted and untargeted agents as well as measurements from the leg. Each paired-agent frame required approximately 1.8 min to acquire, resulting in a total acquisition duration of approximately 63 minutes for the entire dynamic sequence. During this time, T1-weighted MR images of gadolinium uptake were acquired to provide anatomical spatial prior information for the fluorescence tomography reconstruction (example Gd-MRI images for each animal are provided in Figure S1).

After acquisition, volumetric images of fluorescence activity in the head were reconstructed at every frame for each agent such that the processed data consisted of the dynamic uptake in the tumor for each of the two optical agents (as illustrated in Figure 2B-D, and Figure S2) as well as uptake data of both agents in the leg (Figure 2E). Receptor availability estimates for each animal were then obtained using the model-fitting and snapshot RA processing methods, as illustrated in Figure 2F.

### Model-fitting determination of receptor availability

Fitting a paired-agent kinetic model to dynamic uptake data is an established technique for estimating receptor availability 46. This approach is implemented by using a set of differential equations describing the uptake of both targeted and untargeted agents in tissue to derive a single time-dependent expression that depends on the agent kinetics (measured), the receptor availability, and the efflux rate constant of the targeted agent from the plasma to the extravascular space (*k*_2,*T*_):


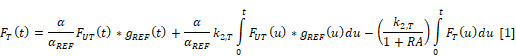


Here, *F_T_*(*t*) and *F_UT_*(*t*) are the measured fluorescence activities of the targeted and untargeted agents, respectively, and the ratio *α/α_REF_* is a calibration factor between targeted and untargeted activities. Notably, this formulation accommodates differences in plasma kinetics between the two agents by deconvolving the agents' uptake with dynamic measurements in a reference tissue assumed to have RA=0, implemented using *g_REF_*(*t*) in the expression above 24. In this study, dynamic measurements from the leg, a commonly-used reference tissue, were used for deconvolution. All variables in the expression were either known or measured except for the parameters *α/α_REF,_ k*_2,*T*_, and RA. These parameters were estimated by fitting the data to Equation 1 using non-linear Levenberg-Marquardt optimization.

### Snapshot receptor availability calculation

Although the model-based approach is a robust approach for determining RA, it requires extensive sampling of the agents over long time periods and expertise in complex model fitting. The snapshot RA approximation is designed to simplify the calculation and reduce the data acquisition requirements to just two frames 47. To compute the snapshot RA at a given time *t*, the fluorescence activities of each agent measured at that time are first normalized to the activities from the first measurement: 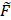
*_T_(t)* = *F_T_*(*t*)/* F_T_*(*0*) and 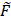
_U*T*_*(t) = F_UT_*(*t*)/* F_UT_*(*0*). Including a plasma uptake correction using fluorescence activity in normal leg muscle, *σ_ref_ (t) =* 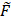
*_UT,Leg_(t)/*
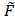
*_T,Leg_(t)* the snapshot RA expression is given by:


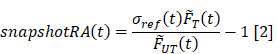


To examine the ability of the snapshot RA to approximate the model-fitting RA in the tumor over time, this parameter was computed for each time frame throughout the dynamic data series.

### Statistical analysis

Receptor availability values for each cohort were compared using one-way analysis of variance (ANOVA) with a post hoc Tukey test for multiple comparisons, and statistical significance was defined as adjusted *p*-value < 0.05. A Shapiro-Wilk test and QQ plot were used to confirm data followed a normal distribution with similar variance (Table S1, Figure S3). Receiver operator characteristic (ROC) curves were determined for treated and untreated groups in each tumor line using the perfcurve function in Matlab (Mathworks, Natick, MA), which determines the true positive rate as a function of false positive rate for different cut-off thresholds, and computes the area under the curve (AUC) values of the resulting curve. Snapshot receptor availability values for treated and untreated animal groups were compared using two tailed t-tests, and a *p*-value < 0.05 was considered statistically significant. Pearson correlation coefficients comparing snapshot and model-fitting RA values were computing using the corrcoef.m function in Matlab.

### Pathological staining and analysis

Shortly after the 24-h MRI-PAFT scans were acquired, animals were euthanized and brain tissues harvested, frozen and later fixed in preparation for pathological analyses. Several 4-μm-thick sections from each specimen were mounted on slides and stained with either H&E or processed for standard IHC staining of EGFR. For the latter, the primary antibody, Abcam #AB52894 (Abcam Inc., Cambridge, MA) was visualized using Leica Bond Refine Detection kit (Cat # DS9800) with a diaminobenzidine (DAB) chromogen and a hematoxylin counterstain. Both H&E and EGFR IHC slides were scanned using a PerkinElmer Vectra3 slide scanner. Estimates of anti-EGFR staining were determined from 20X magnification IHC images using ImageJ (three sites per tumor). The H DAB vector in the Color Deconvolution 1.7 plugin was applied to each image site to deconvolve the EGFR-stain (brown) from the DAB stain. Next, mean intensity values of the EGFR stain channel were computed from all pixels that met a minimum signal intensity criterium determined from the color images. Mean and standard deviation values were then computed for each of the four animal groups.

## Results

### Model-based receptor availability in treated and untreated tumors

**Figure 3:**
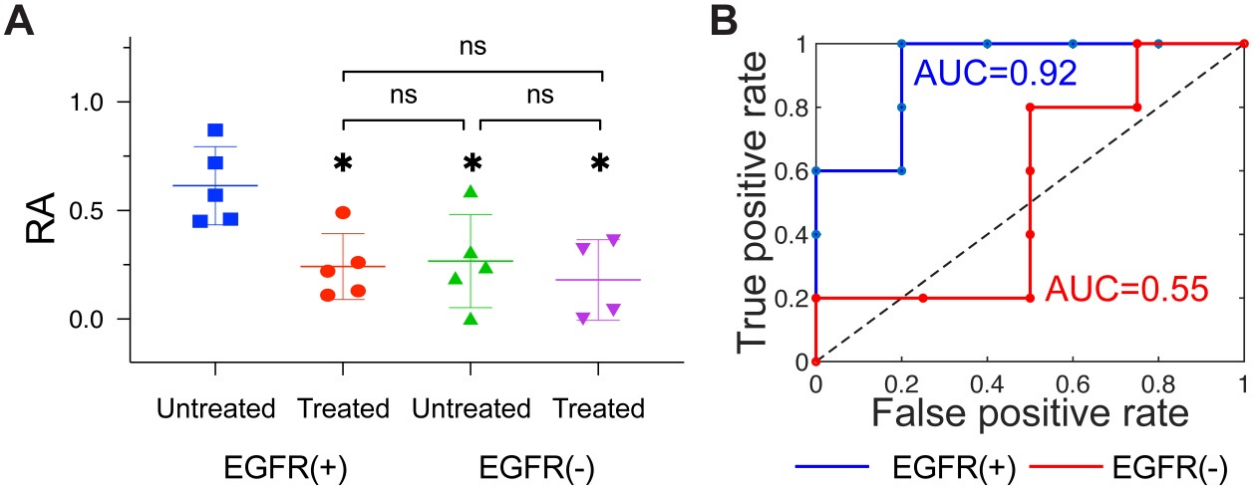
**Receptor availability recovered using the model-fitting approach.** (A): Column scatter plot of RA values for all four groups, with bars showing means and ± standard deviation. Statistical significance was determined using a one-way ANOVA with a post hoc Tukey test (* = *p < 0.05*; **ns** = not significant). The corresponding treated vs. untreated ROC curves for EGFR(+) (blue) and EGFR(-) (red) tumors are shown in (B).

First, we examined the diagnostic performance of the model fitting methodology which uses the entire dynamic sequence to estimate RA in the tumor. The recovered values of RA for treated and untreated EGFR(+) tumors for each animal, as well as mean and quartiles for each group, are plotted in Figure 3A. Mean RA values were 0.24 ± 0.15 and 0.61 ± 0.18 for treated and untreated tumors, respectively, and a one-way ANOVA with post hoc Tukey test confirmed the difference in these means was statistically significant (*p* = 0.027). Receiver operator characteristic analysis comparing treated and untreated groups resulted in the curve in Figure 3B, which yielded an AUC = 0.92, indicating a relatively robust capacity to distinguish these groups. In contrast to the behavior observed in the EGFR(+) group, recovered RA values from the EGFR(-) group, shown in Figure 3C, were similar between treated and untreated groups (RA = 0.18 ± 0.19 and 0.27 ± 0.21, respectively; *p* = 0.89). The corresponding treated vs. untreated ROC curve plotted in Figure 3D provided an AUC = 0.55, showing very limited capacity to distinguish the effects of anti-EGFR treatments. Additional group comparisons showed a statistically significant difference between RA of untreated EGFR(-) and untreated EGFR(+) (*p* =0.041), yet no statistically significant difference between treated EGFR(+) tumors compared to either treated or untreated EGFR(-) tumors (*p* =0.96, and >0.99). A confidence interval analysis further confirmed the statistical significance of these results (Figure S4).

### Single-agent imaging of targeted and untargeted agents

**Figure 4:**
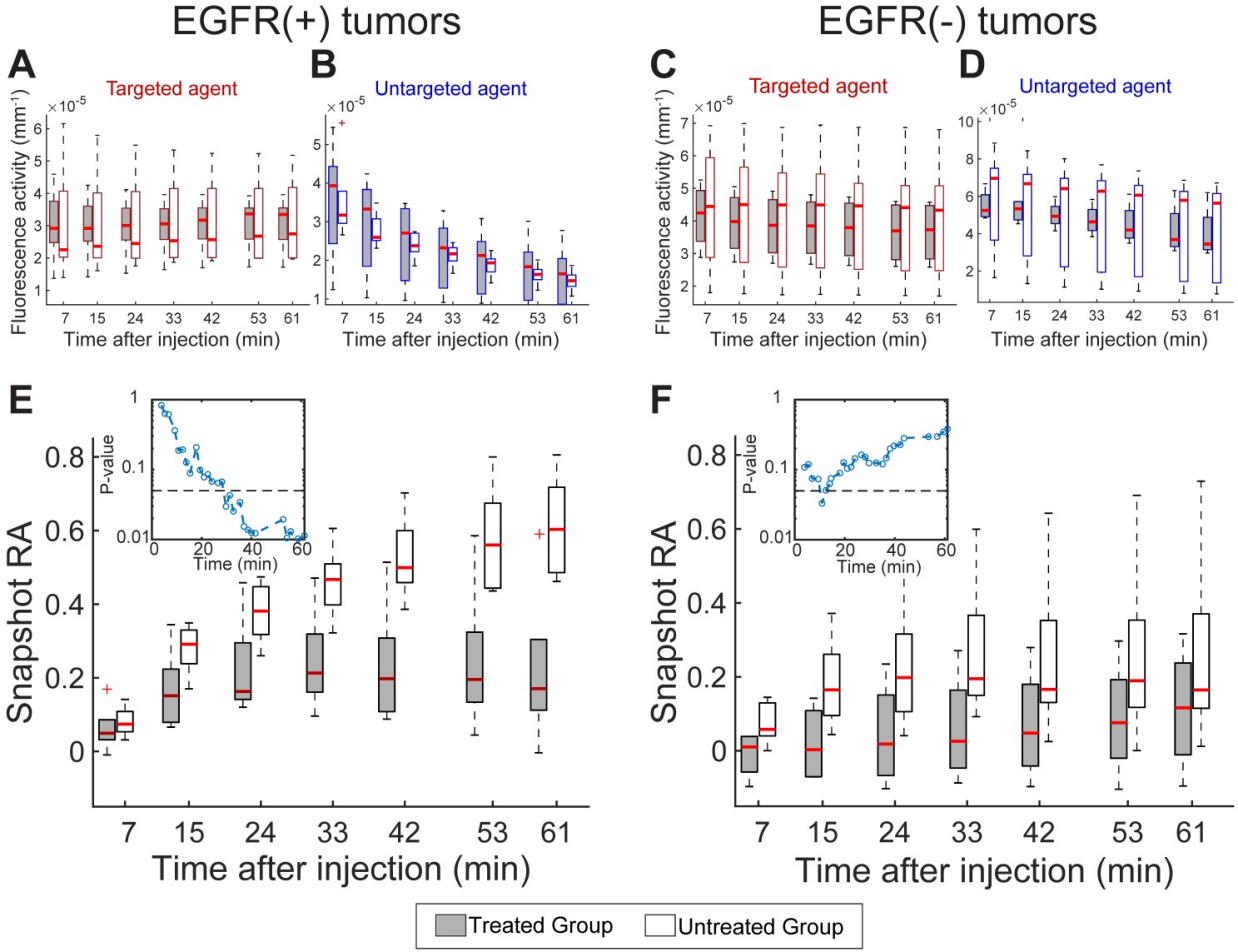
**Single imaging agent uptake kinetics and RA computed using the snapshot approach.** Boxplots (means and quartiles) of targeted (A) and untargeted (B) imaging agent uptake (fluorescence activity) for treated and untreated EGFR(+) tumors computed at select time points during the dynamic imaging sequence. (C) and (D): A similar set of graphs for EGFR(-) tumors. (E) and (F): Snapshot RA group means and quartiles at select time points after agent administration for EGFR(+) and EGFR(-) tumors. The corresponding *p*-values comparing RA means of treated and untreated animals over time are provided for EGFR(+) and (-) tumors in the (E) and (F) insets, respectively. The dashed line in both insets shows *p* = 0.05.

Having established the ability to track RA in response to treatment using the model-fitting approach, we next examined the diagnostic performance of the targeted and untargeted agents alone as well as the snapshot RA values over the course of the dynamic sequence. Figure 4A and B provides the fluorescence activity in EGFR(+) tumors at select time points during the dynamic imaging sequence for the targeted and untargeted agents alone, respectively. Examination of these single-agent uptake curves showed high variability in uptake behavior between animals and no statistical separation between treated and untreated animals at any time point for either agent. These data indicate that the targeted agent uptake in treated animals was elevated compared to untreated. Analogous plots for the EGFR(-) tumors provided in Figure 4C and D also show high variability between animals yet distinct clearance behavior of the untargeted agent compared to the EGFR(+) tumors. These data provide no clear guidance on receptor status or treatment effect.

### Snapshot receptor availability in treated and untreated tumors

The corresponding progression of the snapshot RA values, computed from the targeted and untargeted agent data using Equation 2, are shown in Figure 4E and F for EGFR(+) and (-) tumors, respectively. Unlike the uptake behavior observed for single agents alone, the RA estimates in the EGFR(+) tumor line revealed increasing separation between treated and untreated animals as time progressed. The separation between these groups became statistically significant just 30 min after agent administration, as indicated by the inset* p*-values shown in Figure 4E. With the exception of one early time point, a statistically significance difference in snapshot RA values was not observed between treated and untreated animals with EGFR(-) tumors throughout the dynamic sequence (Figure 4F inset).

### Comparison of snapshot and model-based receptor availability

**Figure 5:**
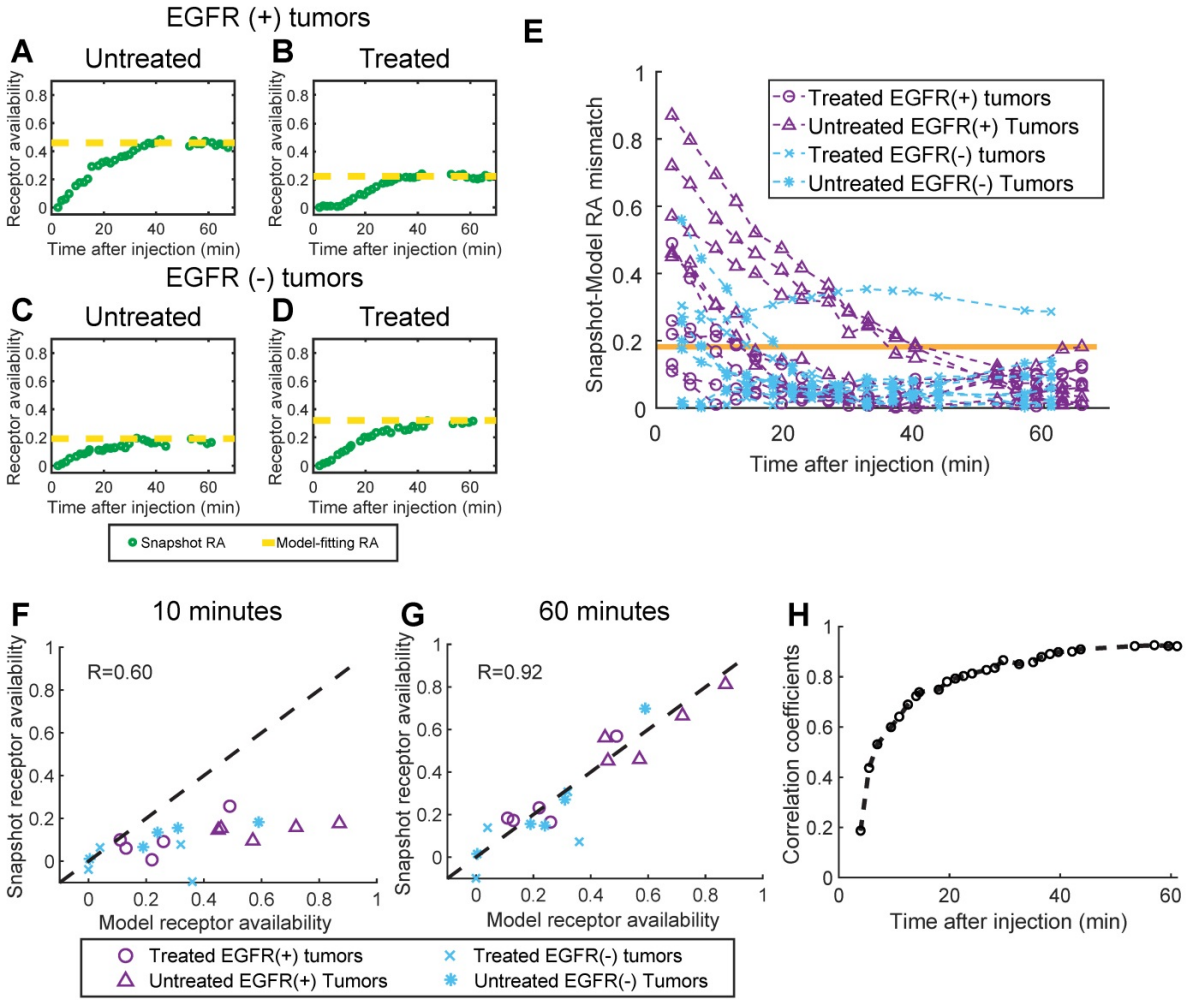
** Comparison of snapshot and model-fitting RA values.** (A)-(D): Snapshot RA values plotted with model-fitting values (dotted line) for four representative animals, one from each tumor line/treatment status group. (E): The absolute difference between the snapshot and model-fitting RA for every animal over time after agent administration. The orange line depicts one standard deviation of the model-fitting RA values for the entire population. (F) and (G): Snapshot RA vs. model-fitting RA plotted for data acquired 10 and 60 minutes after agent administration (dotted line represents unity), from which Pearson's correlation coefficients were determined. Repeating this analysis for every imaging frame in the dynamic sequence and plotting the corresponding correlation coefficients over time yielded the plot in (H), which indicates the snapshot RA values converge to the model-fitting values 30 min after agent administration.

Panels A-D in Figure 5 show snapshot RA values plotted with the corresponding model-fitting RA values for four representative animals (one from each tumor line/treatment group), and suggest that the snapshot approach converges to the model-fitting RA values over the course of the imaging sequence. Inspection of Figure 5E, which provides the absolute difference between the snapshot and model-fitting RA over time, confirms this trend for the population of animals. For all but one animal, the snapshot RA values were within one standard deviation (whole population) of the model-fitting RA values just 40 min after agent administration. Quantifying the correlation between the two methods further confirms the capacity of the snapshot computation to recapitulate the model-fitting values after enough time has elapsed. Figure 5F and G show the correlation plots between model-based and snapshot RA computed at an early (10 min) and late (60 min) time point after administration, respectively. Early in the sequence, the capacity of the snapshot RA to estimate the model-fitting determination is limited (Pearson's correlation coefficient *r* = 0.60), yet by the end of the sequence, the correlation between the two methods is quite high (*r* = 0.92). The temporal progression of agreement between the two methods is further confirmed by examining the model-fitting vs. snapshot RA correlation coefficients for the entire dynamic imaging sequence, plotted in Figure 5H. This analysis revealed that the correlation coefficient exceeded 0.85 after just 30 min, and a high correlation was maintained for the remainder of the sequence (*r* > 0.92 for the last 10 min).

### Single agent imaging 24 h after agent administration

**Figure 6:**
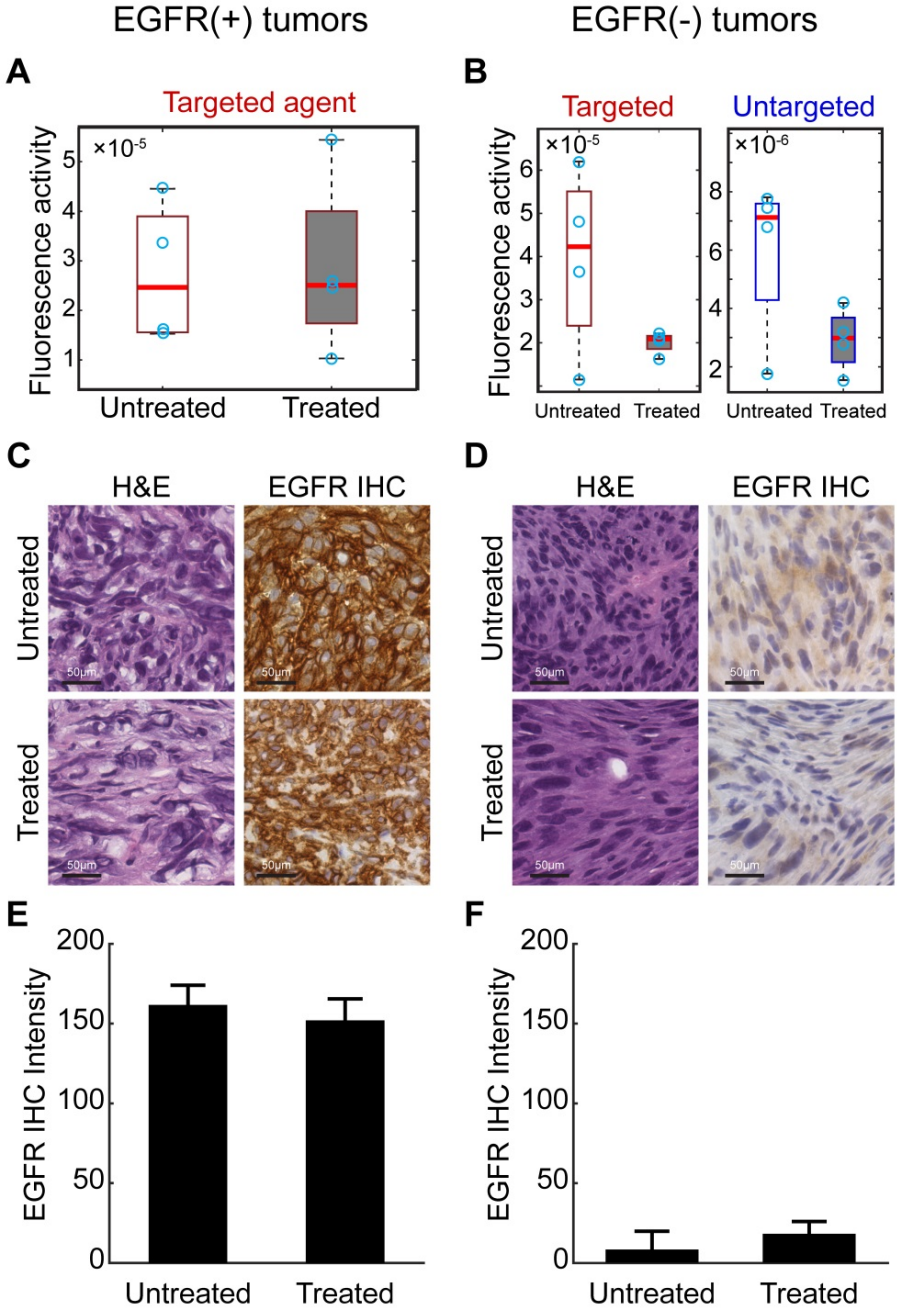
**Long incubation time imaging and pathological analysis.** (A) Recovered fluorescence activity of the targeted imaging agent in the tumor 24 h after administration in EGFR(+) tumors showed no difference between treated and untreated animals. The untargeted agent had washed out and was undetectable in these tumors at this time point-and thus not included in the graphs. (B) Fluorescence activity of targeted and untargeted imaging agents in EGFR(-) tumors 24 h after administration. Although fluorescence activities were different between treated and untreated animals in the targeted channel, the untargeted agent was detectable in these tumors and showed similar behavior, suggesting that non-specific uptake was a primary mechanism of accumulation. (C) and (D) provide representative images of H&E and anti-EGFR IHC slides from excised EGFR(+) and EGFR(-) tumors. Scale bars are 50 μm. Mean and standard deviation of the EGFR IHC stain from all tumors in each group confirm receptor status in each tumor ((E) and (F)).

Waiting for an extended period (24 h) after agent administration before imaging is a commonly-used technique in single-agent molecular imaging to improve receptor-specific contrast. To assess this strategy with the agents in this study, an additional MRI-PAFT imaging session was completed 24 h after agent administration for all animals (i.e. 24 h after the dynamic paired-agent scan was started). Recovered values of tumor fluorescence activity in EGFR(+) and EGFR(-) tumors at this 24 h time point are shown in Figure 6A and B, respectively. In the EGFR(+) tumors, targeted agent tumor uptake showed no capacity to distinguish treated and untreated animals. The untargeted agent had cleared to such an extent that it was largely undetectable (and thus was not included in Figure 6A). In EGFR(-) tumors, the targeted agent accumulation was significantly higher in untreated subjects compared to treated; however, the untargeted agent, present at detectable concentrations, showed similar uptake behavior to the targeted agent, suggesting that non-specific mechanisms were responsible for the observed behavior even at this long incubation time. Finally, the targeted agent alone showed no capacity to distinguish between untreated EGFR(+) and EGFR(-) tumors. These observations suggest that even after long incubation times using these agents, non-specific uptake may still present a major confounding factor challenging binding-specific imaging with single agents.

### Pathology and immunohistochemistry

To confirm the presence of tumor and the retention of EGFR status in each tumor, animals were euthanized shortly after the 24-h imaging session and tumor tissue harvested for H&E and IHC using an anti-EGFR antibody. Representative images of the H&E and IHC stained tissue, and quantitative analysis of the IHC slides are presented in Figure 6C-F. These results confirmed that the EGFR(+) xenografts retained elevated receptor expression while the EGFR(-) line had characteristically low expression. Quantitative analysis of the EGFR IHC images did not show a statistically significant difference in EGFR expression between the cetuximab treated and untreated groups, even in the EGFR(+) tumors. This is not unexpected as the anti-EGFR stain used for IHC targets an intracellular epitope on the receptor, distinct from the extracellular epitope targeted by cetuximab, and the drug does not block the stain from binding the receptor.

## Discussion

The results presented herein are consistent with expectations and provide strong evidence that the noninvasive paired agent approach is capable of assessing RA before and in response to receptor-targeted therapy. Specifically, the study revealed that; (1) the EGFR receptor availability parameter determined using MRI-PAFT provided robust diagnostic performance in discriminating between treated and untreated EGFR(+) tumors, (2) receptor availability values were not significantly different between treated and untreated EFGR(-) tumors, (3) receptor availability values were significantly different between untreated EGFR(+) tumors and EGFR(-) tumors, (4) the snapshot receptor availability technique accurately approximated the more complex model fitting approach as early as 30 min after agent administration, and (5) single-agent imaging of the targeted (or untargeted) agent showed no capacity to discriminate between treated and untreated tumors at any time point, even after long incubation times. Notably, both targeted and untargeted agents accumulated at significantly higher concentrations in EGFR(-) tumors than in EGFR(+) tumors, suggesting that non-specific uptake was a primary determinant in absolute uptake. Yet the paired-agent approach compensated for these effects, stratifying tumors based on expression profile and treatment status. The observed 24 h residual accumulation of the untargeted agent in the EGFR(-) tumor line is a stark indicator of this challenge, suggesting that even at this long time period, non-specific retention can be a dominant source of agent uptake in some tumors. Taken together, these results emphasize the challenges associated with single agent molecular imaging of cancer and show that paired-agent approaches can overcome these barriers to assess receptor availability *in vivo*.

The performance of the snapshot RA calculation was notable, and may help facilitate dissemination and clinical translation of the paired-agent approach. In contrast to the model-fitting approach, which requires extensive dynamic data acquired over a fairly long time period, as well as expertise in multi-parameter data-model fitting, snapshot RA values can be computed through simple ratios from two distinct time points. This relaxes the practical constraints on data acquisition and requirements for technical expertise, making the approach more accessible for preclinical researchers and more readily deployed in the clinic.

The observation that the snapshot paired-agent approach was able to determine RA just 30 min after agent administration has important implications for translational development of the approach. In conventional, single-agent molecular imaging, extending incubation time is generally thought to improve specificity, yet this comes at the cost of the competing effects of signal loss due to agent clearance. The results presented here suggest that development of an appropriate paired-agent methodology, perhaps implemented in a cross-modality construct, could provide rapid assessment of RA, easing the practical constraints of long incubation times used in both clinical and preclinical molecular imaging timing. These short administration times would also facilitate deployment of the approach during fluorescence guided surgery.

An important consideration, however, is the time over which the snapshot RA values converge to the model RA values, which will depend on the pharmacokinetics of the agents used. The agent pair used in the study consisted of fluorescently-labeled Affibody molecules, which are medium-sized molecules (~7 kDa) that display relatively rapid kinetic behavior 31. Thus, accurate RA estimates were obtained within an hour of agent administration. Recovery of RA values using imaging agents that show distribution changes on a longer time scale, such as labeled antibodies 48, will require longer incubation times, though efforts are underway to develop novel agents with optimized molecular weights and clearance rates 49-51.Ongoing efforts in our lab seek to establish a general approach to guide administration intervals for accurate recovery of RA using a range of imaging agents.

Other efforts dedicated to reporting drug-target engagement *in vivo* include the use of fluorescent probes which undergo shifts in fluorescence lifetime to report target activity described in the Introduction, and the use of new PET agents and imaging strategies to reveal target engagement, which are further along the translational path. The latter approaches report changes in signal between pre- and post-treatment scans, using the pre-treatment scans as a reference, and have shown promising results in a small population of patients 15, 52, 53. An important distinction between this approach and the paired-agent methodology is that the latter uses the co-administered untargeted agent as the reference for non-specific accumulation, and thus specific receptor availability can be determined with a single, short imaging session. This could be an important feature in some situations, and alleviates concerns about biological changes between imaging sessions. Nonetheless, the emerging literature support immunoPET strategies are promising and longitudinal monitoring using this approach could be an important clinical tool in the future.

Although discussed herein using optical imaging, the principles behind the quantitative paired-agent strategy are generally applicable to other imaging modalities, provided multiple agents can be imaged simultaneously. This prerequisite is fulfilled using fluorescent agents with distinct emission spectra; however, the use of optics for non-invasive imaging deep in tissue, especially in larger tissues volumes, can be limiting. The fluorescence tomography approach studied here is subject to sensitivity that is strongly depth-dependent and produces relatively low resolution images due to photon scatter. In this context, the potential for paired agent approaches using optics in the clinic will generally fall into the following categories: (1) diagnostic imaging of superficial tissue accessible directly or through endoscopy/implants, (2) intra-surgical applications, and (3) fluorescence tomography of shallow (centimeter-scale) sub-surface pathologies in accessible tissue volumes, such as in shallow intracranial tumors.

Enabling the paired-agent technique to report receptor availability anywhere in the human body will require the use of imaging modalities capable of deep-tissue imaging, such as PET, CT and MRI. Although most of these technologies are incapable of multiplexed imaging of more than one agent at a time, new chemical exchange saturation transfer (CEST) MRI techniques may enable paired-agent imaging in larger tissue volumes using MRI 54, 55, and a recent study reported on multi-agent ratiometric imaging using SPECT tracers 56. Ongoing studies in our lab suggest that with proper calibration, co-registration and uptake deconvolution, it will be possible to deploy paired agent imaging using a cross-modality strategy. In this paradigm, a targeted agent, imaged using PET, for example, could be paired with a reference agent imaged using CT or MRI to produce maps of quantitative receptor imaging in patients. Validation of this novel, multi-modality approach, founded upon the principles shown herein, would represent a major advance for molecular imaging, and enable the application of this quantitative receptor imaging approach throughout clinical medicine.

The lack of existing techniques to assess receptor availability *in vivo* poses a challenge for direct validation of the MRI-PAFT strategy evaluated here. However, the use of multiple well-characterized tumor lines with different properties and an array of independent measurements supports the central hypothesis. Flow cytometry and IHC assays have routinely shown that U251 cells have elevated expression of human EGFR, while 9L cells have undetectable levels of human EGFR 26. Accordingly, extensive inhibition studies *in vitro* have shown that cetuximab blocks binding of the targeted probe (ABY029) in U251 cells, but has no effect in 9L cells 26, 27. Importantly, the results herein, which show low RA for treated and untreated 9L tumors and statistically significant differences in RA for treated and untreated U251 tumors, are fully consistent with these prior reports. Additionally, IHC for each xenograft used in the study confirmed that the expected expression profiles were retained, and the results were not due to an overall loss of EGFR in treated animals. Notably, the IHC probes used target an internal binding domain on the receptor, and are not affected by the extracellular binding of cetuximab or the imaging probe. Finally, we note that several previous studies using invasive techniques have confirmed in multiple tumor lines that paired agent estimation of RA correlates strongly with receptor expression 23, 46. Taken together, the results are consistent with expectations based on independent measurements.

The results reported herein show that noninvasive paired-agent imaging can accommodate the confounding effects of nonspecific uptake to quantify receptor availability in response to receptor-targeted therapy in cancer, a notoriously difficult parameter to measure in solid tumors. Recovery of this important metric can be used to determine; (1) whether the target is present and available in abundance before treatment and (2) whether the drug binds to the targeted receptor, effectively decoupling target accessibility from downstream activity. This capability is readily applicable to preclinical imaging studies for developing and evaluating receptor-targeted therapies, as demonstrated here, and offers a promising path towards clinical translation through the development of cross-modality techniques. Deployment of these strategies for routine assessment of drug targets in patients could have a profound impact on guiding patient-specific treatment regimens.

## Supplementary Material

Supplementary figures and tables.

## Abbreviations

MRI: Magnetic resonance imaging
EGFR: epidermal growth factor receptor
RA: receptor availability
MRI-PAFT: MRI-coupled paired-agent fluorescence tomography
H&E: hematoxylin & eosin
IHC: immunohistochemistry
IACUC: Institutional Animal Care and Use Committee
Gd: gadolinium
ANOVA: analysis of variance
ROC: receiver operator characteristic
AUC: area under the curve
DAB: diaminobenzidine
