# Supplementary material for: Noninvasive quantification of target availability during therapy using paired-agent fluorescence tomography
Source: Theranostics. 2020 Sep 14;10(24):11230–43. doi: 10.7150/thno.45273 (PMC7532673; doi:10.7150/thno.45273)
Supplement: Supplementary file 1 — Supplementary figures and tables. [file thnov10p11230s1.pdf]

Supplementary Information

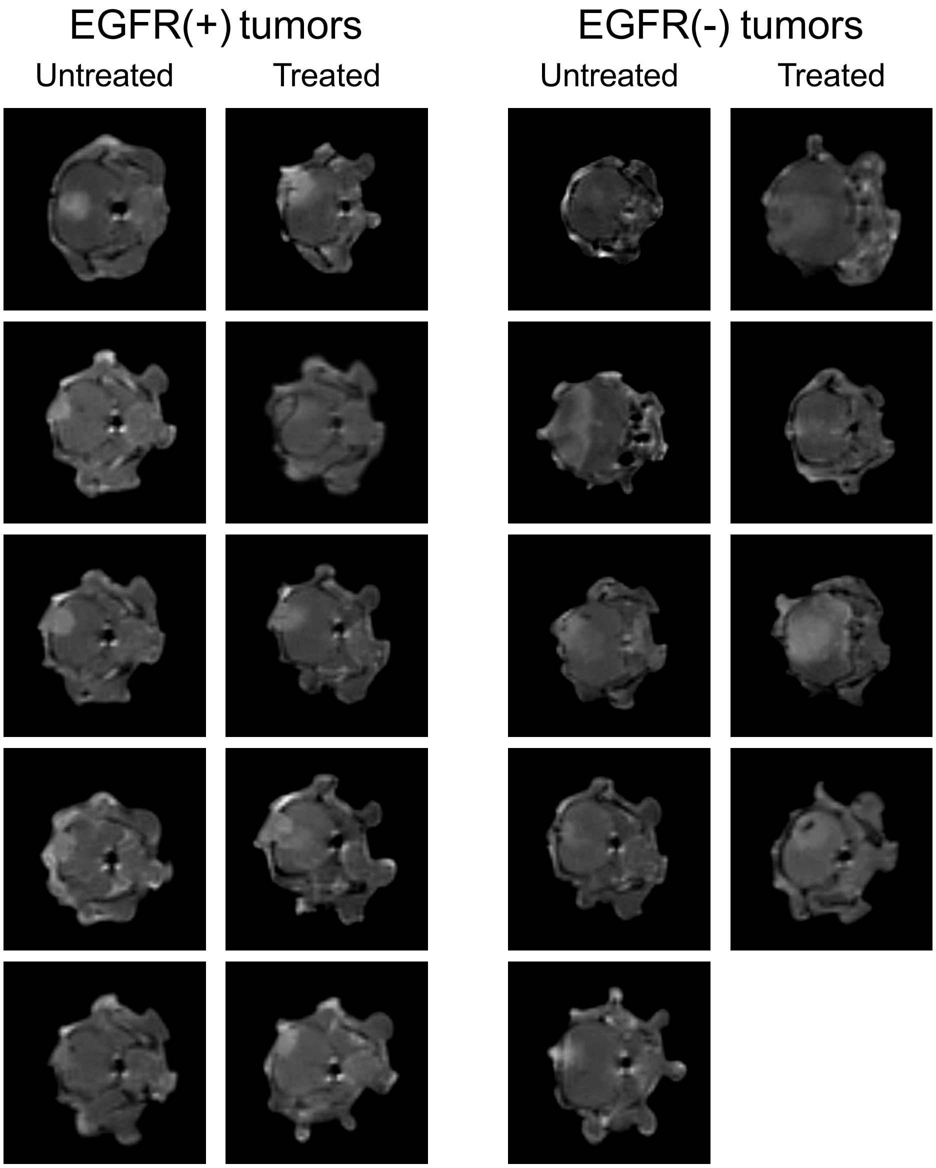

Figure S1. Head scans of gadolinium-enhanced T1-weighted MR images for each animal included in the study (one slice per mouse shown). Indentations in the tissue show the locations of the optical fibers.

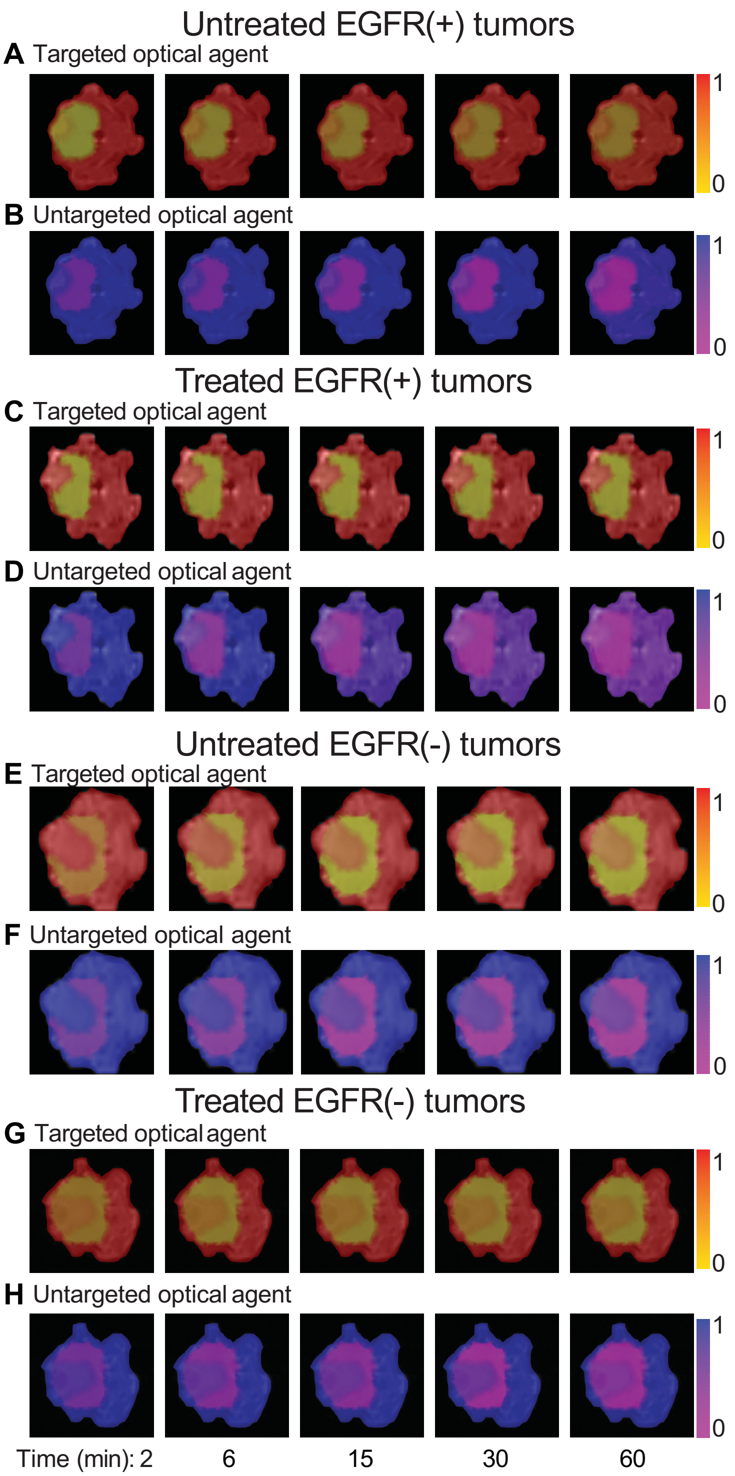

Figure S2. Recovered values of fluorescence yield (targeted and untargeted channels) overlaid on MRI scans for four animals (one from each group). All reconstructions were done using the hard-priors technique in 3-D and a slice extracted for visualization. Selected frames over the sequence are shown. Values from the tumor were extracted and used in the paired-agent models to determine RA.

Testing data distribution normality:

To confirm the RA data follow a normal distribution, we performed a Shapiro-Wilk test as well as a Q-Q (quantile-quantile) plot. Shapiro-Wilk test statistics and p-values are shown in Table S1, and indicate that all cohorts show p-values above the alpha level ($\alpha = 0.05$). Thus, the test was unable to reject the null hypothesis that the data populations are normally distributed. The Q-Q plot is provided in Figure S1 and shows strong similarity between experimental distributions and theoretical Gaussian distributions for all cohorts (Figure S1).

Table S1. Shapiro-Wilk normality test of RA distributions for each experiment cohorts

Shapiro-Wilk test	EGFR(+) Treated	EGFR(+) Untreated	EGFR(-) Treated	EGFR(-) Untreated
**W**	0.8737	0.9039	0.8311	0.9620
**P value**	0.2816	0.4320	0.1706	0.8220
**Passed normality test (alpha=0.05)?**	Yes	Yes	Yes	Yes

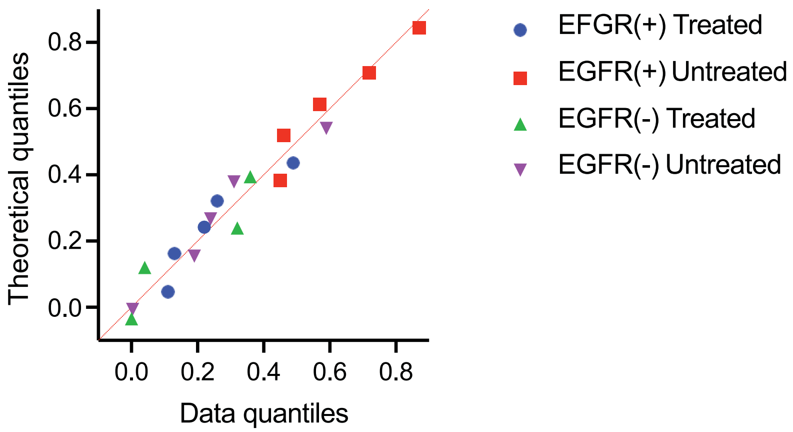

Figure S3. Normal Q-Q plot of RA distributions.

Further analysis of Confidence Intervals:

To correct for familywise error rate in multiple comparison procedure (MCP), a Tukey post hoc correction was applied for one-way ANOVA analysis. Test result indicates statistically significant in one-way ANOVA test. Assuming normal distribution, 95% Confidence Interval analysis with Tukey correction of differences in the means further confirms the test results (Figure S2)

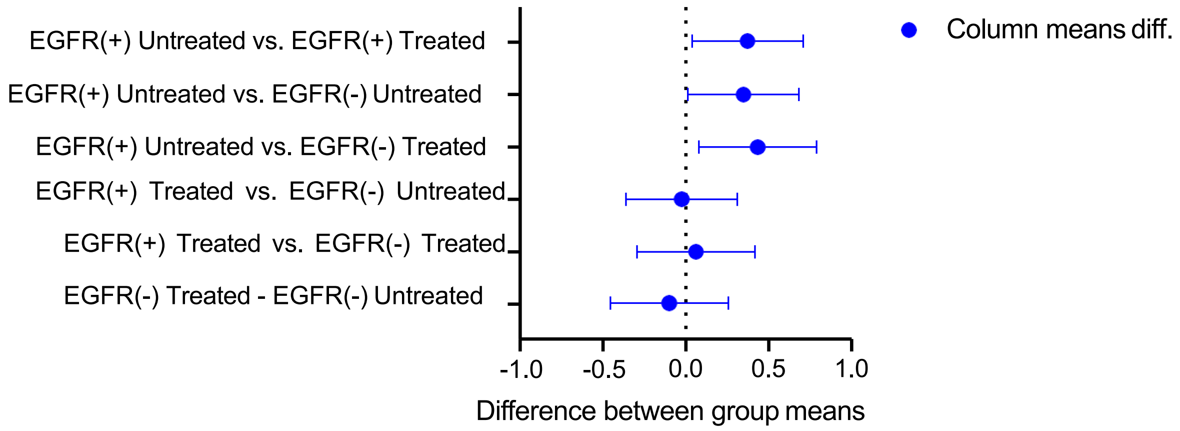

Figure S4. 95% Confidence Interval plot showing the difference between group means for multiple comparisons.
